# Bat white-nose disease fungus diversity in time and space

**DOI:** 10.3897/BDJ.12.e109848

**Published:** 2024-02-02

**Authors:** Violeta L Zhelyazkova, Nicola M. Fischer, Sebastien J Puechmaille

**Affiliations:** 1 National Museum of Natural History, Bulgarian Academy of Sciences, Sofia, Bulgaria National Museum of Natural History, Bulgarian Academy of Sciences Sofia Bulgaria; 2 ISEM, University of Montpellier, CNRS, EPHE, IRD, Montpellier, France ISEM, University of Montpellier, CNRS, EPHE, IRD Montpellier France; 3 Zoological Institute and Museum, University of Greifswald, Greifswald, Germany Zoological Institute and Museum, University of Greifswald Greifswald Germany; 4 Institut Universitaire de France, Paris, France Institut Universitaire de France Paris France

**Keywords:** Chiroptera, emerging infectious disease, fungal pathogen, wildlife disease, white-nose syndrome, Leotiomycetes, Thebolales, Pseudeurotiaceae

## Abstract

White-nose disease (WND), caused by the psychrophilic fungus *Pseudogymnoascusdestructans*, represents one of the greatest threats for North American hibernating bats. Research on molecular data has significantly advanced our knowledge of various aspects of the disease, yet more studies are needed regarding patterns of *P.destructans* genetic diversity distribution. In the present study, we investigate three sites within the native range of the fungus in detail: two natural hibernacula (karst caves) in Bulgaria, south-eastern Europe and one artificial hibernaculum (disused cellar) in Germany, northern Europe, where we conducted intensive surveys between 2014 and 2019. Using 18 microsatellite and two mating type markers, we describe how *P.destructans* genetic diversity is distributed between and within sites, the latter including differentiation across years and seasons of sampling; across sampling locations within the site; and between bats and hibernaculum walls. We found significant genetic differentiation between hibernacula, but we could not detect any significant differentiation within hibernacula, based on the variables examined. This indicates that most of the pathogen’s movement occurs within sites. Genotypic richness of *P.destructans* varied between sites within the same order of magnitude, being approximately two times higher in the natural caves (Bulgaria) compared to the disused cellar (Germany). Within all sites, the pathogen’s genotypic richness was higher in samples collected from hibernaculum walls than in samples collected from bats, which corresponds with the hypothesis that hibernacula walls represent the environmental reservoir of the fungus. Multiple pathogen genotypes were commonly isolated from a single bat (i.e. from the same swab sample) in all study sites, which might be important to consider when studying disease progression.

## Introduction

Genetic diversity not only provides the raw material for the evolution of species, but significantly influences the size, dynamics and fitness of a population, species interactions and ecosystem functions (Hughes et al. 2008). It also plays a crucial role in pathogen-host interactions, virulence and transmission and determines the potential for the emergence of more (or less) adaptative and dangerous pathogen variants. Thus, information on a pathogen’s genetic diversity and its distribution is important for understanding infectious diseases, including wildlife diseases such as White-nose disease (WND) in hibernating bats.

White-nose disease represents one of the greatest challenges for North American bat conservation. The disease has caused an estimated 5.7 to 6.7 million casualties for the first 5 years after its emergence (White Nose Response Team 2011) and the colonies of the three most affected species have suffered up to more than 90% population declines (Cheng et al. 2021). Although the WND causative agent, the fungus *Pseudogymnoascusdestructans* (*Blehert et al. 2009*) can be visually recognised (Fritze et al. 2021), molecular tools are widely used since the beginning of WND research. The pathogen was first characterised as a novel species by combining morphological and genetic data (Gargas et al. 2009); soon after, genetic data were used to report its presence in Europe (Puechmaille et al. 2010). Based on patterns of genetic diversity and similarity (Rachowicz et al. 2005), Europe was quickly identified as the source population from where *P.destructans* in North America was introduced (Leopardi et al. 2015, Drees et al. 2017b). This explains the pervasive mortality due to WND in the naïve North American species and the lack of mass morbidity or mortality in European species that co-evolved with the fungus (Puechmaille et al. 2010, Puechmaille et al. 2011a, Puechmaille et al. 2011b, Zukal et al. 2016, Fritze and Puechmaille 2018). Subsequently, large sections of WND research, especially on fungal load and identification of *P.destructans* (e.g. Chaturvedi et al. 2011, Muller et al. 2012, Shuey et al. 2014, Niessen et al. 2022), have relied on molecular tools. Yet, more studies are needed to better characterise patterns of *P.destructans* genetic variation at large scale (continental-wide) and quantify dispersal/gene flow between established populations (though see Forsythe et al. 2021, Fischer et al. 2022).

At small geographical scales (within individual hibernacula), the use of genetic data has been central in elucidating *P.destructans* life cycle and transmission routes. Indeed, Fischer et al. (2022) demonstrated that bats become infected with *P.destructans* anew each autumn when they return to their hibernacula, acting as a long-term reservoir of the fungus (see also Puechmaille et al. 2011a, Hoyt et al. 2021, Hicks et al. 2023). During the winter, the pathogen undergoes clonal reproduction on its hosts and large amounts of spores are shed back into the reservoir in late hibernation. During the summer, bats clear *P.destructans* and the cycle is repeated the next year (see also Hoyt et al. 2021, Hicks et al. 2023). However, many questions remain regarding *P.destructans* levels of genetic diversity and its distribution within a site, such as: Is the population homogeneous within a site, suggesting ample pathogen movement? Or are there any factors acting as barriers for *P.destructans* spread at small spatial scales? Are patterns of genetic diversity distribution similar across sites? Do bats become infected by multiple fungal individuals?

To address these questions, we intensively studied *P.destructans* at three bat hibernacula in Europe: two karst caves in Bulgaria and a disused cellar in Germany (cf. Fischer et al. (2022)). We applied the fine-scale approach developed by Fischer et al. (2022), whereby fungal individuals are genetically distinguished as multilocus genotypes (MLGs) and tracked through time and space. This allowed us to test for genetic differentiation between and within sites, the latter being the main goal of the study. Within sites, we tested for differentiation across years and seasons of sampling; across sampling locations within the site (rooms); and between bats and site walls. We also calculated the size of fungal populations for each site, estimated the frequency of multiple infections (involving different MLGs) in bats and interpreted our results in the light of host-pathogen interactions and disease transmission dynamics. We expected that genetic differentiation would be much stronger between than within sites; that genetic diversity would be variable between sites, but within the same order of magnitude; and that multiple infections in bats would be common in all sites.

## Materials and methods

### Sample collection

For Bulgaria, swab samples were collected from bats and walls in two karst caves, Balabanova dupka (N43.13, E23.04) and Ivanova voda (N41.89, E24.88; Suppl. material 1), representing two of the largest known hibernacula for *Myotismyotis*/*blythii* in the Balkans. Around 2500 *M.myotis*/*blythii* hibernate in Balabanova dupka, (personal data), while a maximum of 5,600 *M.myotis*/*blythii* and 2,500 *Myotiscapaccinii* hibernate in Ivanova voda (EUROBATS 2011). We used the species complex *M.myotis*/*blythii* as differentiating the two species during hibernation is challenging without handling.

Swab samples were collected from the muzzle, ears or wings of freely hanging and visibly infected *M.myotis*/*blythii* at the end of the hibernation season (March – June) between 2015 and 2019, without handling the bats (Fritze et al. 2021). Bats with visible fungal growth are only present during this period. Depending on the number of visibly infected body parts, one or more swabs were collected from each bat, i.e. one per infected body part. Infection is herein defined as the acquisition of a microbe by a host (Casadevall and Pirofski 2000). Additionally, wall swabs were collected both at the end of the hibernation season (March – June) and prior to its start (October). These were taken either in close proximity (a few centimetres) to the main bat roosting places or further away from them (around 50 metres, see Suppl. material 2). For accuracy, we used plastic markers that stayed in the caves for the full period of the study and we collected four swabs at 1 metre up, 1 metre down, 1 metre left and 1 metre right of each marker. Each swab touched the cave wall nine times covering an area of approximately 10 сm^2^. Within each site, sampling locations were distributed in different rooms as presented in Suppl. material 2. Both bat and wall swabs were then placed in sterile Eppendorff tubes and were transported to the lab on the same day. In the lab, samples were stored in the fridge until plating (usually a few days after collection).

For Germany, we used data collected in the same way over 5 years (2015–2019) from the Eldena hibernaculum (N54.09, E13.44) situated in the north-eastern Federal State of Mecklenburg-Vorpommern (Suppl. material 1). The site is described in detail in Fischer et al. (2022). Briefly, it is a disused beer cellar with 12 rooms, where a total of 300-400 bats hibernate, mostly *Myotisdaubentonii*, *M.nattereri* and *M.myotis*, the latter being the most commonly infected species. A map of the site is presented in Suppl. material 3.

### Cultures

We used culturing methods to physically separate different fungal genotypes infecting the same host, to provide a sufficient quantity of fungal material for the microsatellite analysis and to prove that the studied fungal spores were viable. The percentage of *P.destructans* positive samples per season varied between 3 and 74 for samples collected from the cave walls (*P.destructans* not visible) and between 42 and 100 for samples collected from bats (*P.destructans* visible). The lowest values for wall samples came from Ivanova voda where spring floods regularly occur, potentially washing away or killing fungal spores. Another factor that can reduce fungal yield is the time elpased between sample collection and plating: in 2017, due to the lack of funding, we plated some samples later than in other years.

In the lab, each swab was cultured on a Petri dish containing DPYA growing medium (Vanderwolf et al. 2016a). Plating was done by tapping the swab five times at the centre of the dish, then adding 0.1 ml of distilled water (dH_2_O) and spreading it with a sterile metal spatula. Plates were monitored daily for growth and individual spores (i.e. single spores) were physically separated and transferred into new Petri dishes as soon as they were visible (as described in Fischer et al. 2022). New plates containing single spore isolates were then sealed with parafilm and stored upside down in an incubator at 10°C for at least three months until material was harvested for DNA extraction. During this time, they were regularly monitored for the growth of other fungal species (that were physically removed when possible) or for the growth of more than one *P.destructans* spore. Contaminated cultures or cultures that did not originate from a single *P.destructans* spore were excluded from the analysis. For consistency, we limited the number of single spore isolates obtained from a single swab sample to six. Thus, we did not isolate all fungal spores in a given swab, but instead, we aimed to study the entire *P.destructans* genetic diversity within a site by using a large number of samples.

### Molecular analyses

DNA extraction was performed following the protocol in Fischer et al. (2022). We then genotyped *P.destructans* isolates using 18 microsatellite markers (Drees et al. 2017a) and two mating type markers in four PCR multiplexes as described in Dool et al. (2018), see also Fischer et al. (2022). Genotyping was carried out using an ABI 3130 Genetic Analyser (Applied Biosystems) and the GeneMapper Software v.5 (Applied Biosystems).

### Data analysis

The genotypic analysis was based on the identification of multilocus genotypes (MLGs) which are defined by the distinct combination of alleles at the 18 microsatellite loci. Despite the presence of two mating types in this species (Palmer et al. 2014), sexual reproduction has never been observed and a 4-years longitudinal study at one site in Germany provided strong evidence that clonal genotypes were omnipresent in *P.destructans* (see Fischer et al. 2022). Therefore, as already demonstrated by Fischer et al. (2022), MLGs of this haploid and the clonally reproducing organism can be used to track fungal individuals in space and time. Thus, detection of the same MLG in different hibernacula or in different substrates/rooms means that *P.destructans* has moved between those.

Missing data were not used as information to define MLG identity and MLGs containing more than 20% of missing data (seven MLGs in total) were excluded from the analysis. Calculations of allelic diversity and differentiation were performed on clone-corrected data, meaning that only one single spore isolate of each MLG was retained per site or per analysed within-site groups. All analyses were performed in R software (version 4.0.1, R Core Team 2019).

### Population differentiation

To estimate population structure between and within hibernacula, we tracked individual MLGs in space and time. To evaluate MLG movement, we divided the number of shared MLGs between the factors considered (site, hibernaculum room, year and season of sampling (winter-spring/autumn) and substrate (bat/wall)) by the total number of MLGs (i.e. the total number of MLGs across sites [for the factor site] or total number of MLG per site [for other factors]). We removed MLGs appearing only once throughout the dataset as, by definition, these cannot be shared.

We also applied AMOVA (analysis of molecular variance, Excoffier et al. 1992) based on allele frequencies, with 1000 permutations to test for significance (allelic differentiation). The factors analysed by the AMOVA were: site, hibernaculum room, year and season of sampling (winter-spring/autumn) and substrate (bat/wall). All factors were analysed both separately and in combination and all tests were carried out twice, with (using a 5% threshold) and without removing loci containing missing data. When testing allelic differentiation between rooms, the rooms with a number of swab samples below 10 were excluded (see Suppl. material 5). Given that both genotypic differentiation (based on MLGs) and allelic differentiation (based on allele frequencies) are proxies for genetic differentiation, we hereafter use the term ‘genetic differentiation’ to refer to both measures.

### Genotypic richness and population size

The number of expected MLGs (eMLGs) at the smallest shared sample size (based on rarefaction with 1000 permutations) was used to compare genotypic richness (total number of MLGs) between hibernacula. For estimating population size (= expected genotypic richness or expected total number of MLGs), we used the CMRPopHet functions implementing a capture-mark-recapture (CMR) model, based on a single sampling event (Petit and Valiere 2006). This model assumes that each MLG has the same probability of being sampled, meaning that all MLGs should have the same relative frequency of occurrence within the population. In order to test this assumption, we used the heterogeneity test developed by Puechmaille and Petit (2007). Considering that the exact number of single spores isolated per swab was not identical, we validated that the sampling design/intensity did not influence our estimates by running the analyses after randomly selecting exactly one single spore isolate per swab (run 1000 times to account for stochastic effects).

### Environmental reservoir and infections in bats

By comparing genotypic richness of the fungus isolated from bats and from hibernaculum walls, Fischer et al. (2022) demonstrated that yearly re-infection of bats originates from hibernaculum walls (the environmental reservoir) containing viable spores of *P.destructans*. To corroborate these results, we tested the expectation that a transmission bottleneck would exist between the reservoir and the hosts, whereby genotypic richness of the pathogen would be reduced on the hosts as not all MLGs are successfully transferred from the source to the host population. Therefore, we compared genotypic richness of the fungus isolated from bats and from hibernacula walls by estimating the number of eMLGs at the smallest shared sample size, based on rarefaction with 1000 permutations. We additionally ran the analyses when randomly selecting exactly one single spore isolate per swab and when randomly selecting exactly two single spore isolates per swab (run 1000 times). To compare the genotypic richness per wall or bat swab, we calculated the following ratio for each swab: (G-1)/(N-1), where G is the number of MLGs per swab and N is the number of single spore siolate per swab (Dorken and Eckert 2001). This ratio varies between 0 when all isolates from a sample harbour the same MLG to 1 when all isolates from a sample harbour a different MLG. The average of this ratio was calculated per swab type (wall versus bat). To estimate the frequency of multiple infections in bats, we calculated the percentage of cases where more than one MLG or more than one mating type was found on a single bat swab, after taking exactly three single spore isolates per swab.

## Results

We used here a dataset containing a total of 1925 *P.destructans* single spore isolates, of which 608 came from Balabanova dupka, 255 from Ivanova voda and 1062 from Eldena. The average number of single spore isolates per bat swab was 4.84 (range 2-6, median 5) for Balabanova dupka, 4.5 (range 1-6, median 5) for Ivanova voda and 2.76 (range 1-4, median 3) for Eldena. For wall swabs, we obtained an average of 2.78 single spore isolates per swab (range 1-6, median 2) for Balabanova dupka, 2.21 (range 1-6, median 1) for Ivanova voda and 3.51 (range 1-5, median 4.5) for Eldena. We successfully amplified all microsatellite and mating type markers, with an overall amount of missing data of 1.2%.

### Population differentiation

**Figure 1.**
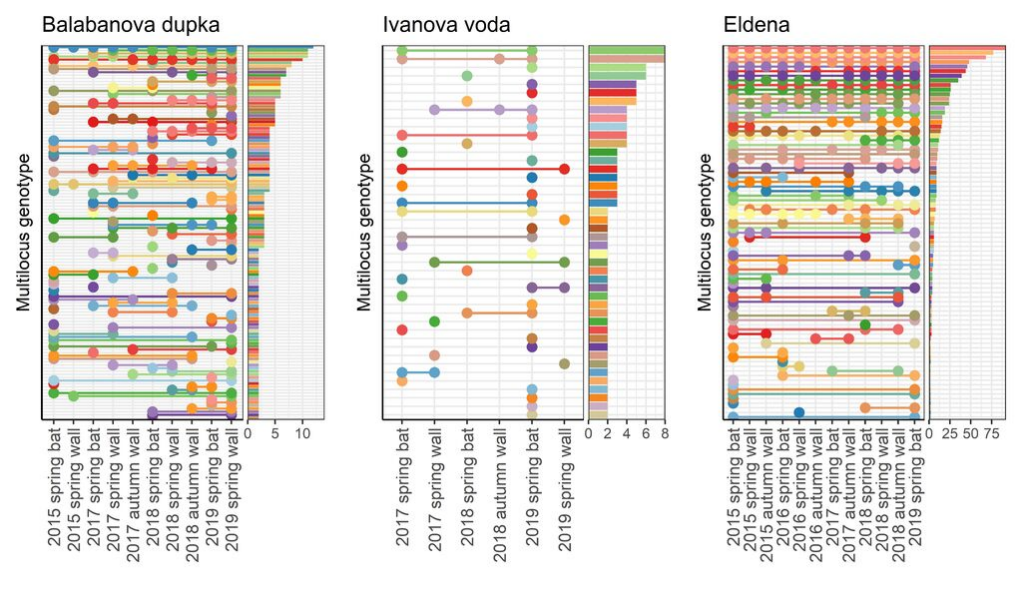
Visual representation of shared multilocus genotypes (MLGs) in the three study sites Balabanova dupka, Ivanova voda and Eldena across time and between bats and walls. Each row represents a particular MLG and a circle signifies that this MLG was detected during the particular sampling event. The bar graph represents the relative frequencies of MLG occurrence. The code used to recreate the graph was obtained from Kamvar et al. (2015). For convenience, only MLGs that were detected more than once are included on the graphs.

The AMOVA detected no significant allelic differentiation within sites with regard to any of the factors considered: hibernaculum room, time of sampling (year and season) and substrate (bat or wall). Additionally, within a site, MLGs were often shared amongst rooms, years and season and substrates, especially in Balabanova dupka and Eldena. Across hibernaculum rooms, 54.2%, 6.8% and 80.2% of MLGs were shared in Balabanova dupka, Ivanova voda and Eldena, respectively, including rooms where bats were not encountered during our hibernation surveys. Across sampling years and seasons, 61.7%, 22.7% and 80.2% of MLGs found at least twice were shared in Balabanova dupka, Ivanova voda and Eldena, respectively (Fig. 1). Shared occurrences were encountered both in the presence (in winter-spring) and absence (in autumn) of hibernating bats with visible fungal infection. Between bats and walls, 45%, 11.4% and 65.4% of MLGs were shared in Balabanova dupka, Ivanova voda and Eldena, respectively (Fig. 1).

As shown by AMOVA (see Suppl. materials 4, 5 for sample sizes), there was significant allelic differentiation amongst sites explaining 26.88% of total variance (p < 0.001). Results were the same with or without removing missing data. The strong allelic differentiation was corroborated by the genotypic differentiation showing that none of the 615 *P.destructans* MLGs detected was shared amongst any of the three study sites.

### Genetic diversity and mating types

All studied loci were polymorphic, their mean richness being highest in Ivanova voda and lowest in Eldena (Table 1). Within sites, allelic richness widely varied per locus (range: 2-64 alleles; Suppl. material 6). A total of 301 unique MLGs were obtained from Balabanova dupka, 165 from Ivanova voda and 149 from Eldena. At equal sample size, genotypic richness was more than two times higher in Balabanova dupka and Ivanova voda compared to Eldena (Table 1). Differences in allelic and genotypic richness between the study sites were consistent when we repeated the analysis with only one single spore isolate per swab (Suppl. material 8).

Within sites, genotypic richness of *P.destructans*, estimated as the number of eMLGs, was consistently higher in swab samples taken from walls than in swab samples taken from bats (Table 2). Results were consistent (bar one exception) when we repeated the analysis with one single spore isolate per swab or two single spore isolates per swab (Suppl. material 9). The mean genotypic richness per swab, estimated as (G-1)/(N-1), was also higher in wall swabs (Table 2).

Multiple MLGs were very commonly found on the same bat swab. Indeed, after taking exactly three single spore isolates per swab, multiple MLGs were found in 83.6%, 80.9% and 83.3% of bat swabs in Balabanova dupka, Ivanova voda and Eldena, respectively. Both mating types of the pathogen were found in 44%, 39.3% and 46.4% of bat swabs in Balabanova dupka, Ivanova voda and Eldena, respectively.

Both mating types were present in all hibernacula although with different proportions, whereby the Bulgarian sites were more similar to each other than they were to the German site (Table 1).

### Population size estimates

According to the CMR model, *P.destructans* population size (or the total number of MLGs predicted to be present) was 377 MLGs (Highest Probability Density [HPD] 95% 352-401) for Balabanova dupka, 274 MLGs (HPD95% 233-317) for Ivanova voda and 150 MLGs (HPD95% 150-151) for Eldena (Table 1). Although moderate, some heterogeneity was observed in the probability of MLG sampling for Balabanova dupka and Ivanova voda, while a seemingly stronger heterogeneity was present in Eldena (Suppl. material 7).

The dataset and the R script used for the analysis are presented in Suppl. materials 10, 11, respectively.

## Discussion

### Lack of significant P.destructans genetic differentiation within sites

The present study is the first one to characterise *P.destructans* genetic diversity and its distribution at multiple spatial scales in natural hibernacula within the native range of the fungus. As expected, none of the factors considered within a site, including substrate (bats or walls), time (across sampling years and seasons) or space (hibernaculum rooms), was associated with significant allelic or genotypic differentiation of *P.destructans*. The lack of significant differentiation between bats and hibernaculum walls is consistent with the known life cycle of *P.destructans*, whereby yearly re-infection of bats originates from the environmental reservoir, which is, in turn, replenished by bats shedding fungal spores towards the end of the hibernation season (Fischer et al. 2020, Hoyt et al. 2021, Fischer et al. 2022, Hicks et al. 2023). While the presence of fungal DNA on active bats during the summer has been documented, it remains at a relatively low prevalence, estimated at below 1% (Hoyt et al. 2021). However, it is important to note that positive quantitative PCR (qPCR) data alone do not offer insights into spore viability, a crucial factor in understanding disease dynamics. Therefore, interpreting the meaning and implications of qPCR-positive bats during the summer is challenging. Culture-based studies have shown that a few individual bats carry viable fungal spores in the summer (Dobony et al. 2011, Ballmann et al. 2017). Nevertheless, evidence suggests that these are rare and likely result from de novo infections rather than carry-over from the previous hibernation period (discussed in Fischer et al. 2021).

The lack of significant differentiation across sampling seasons is consistent with the long-term survival of the pathogen in hibernacula, demonstrated by Fischer et al. (2020) and Fischer et al. (2022) and previously suggested in other studies (e.g. Puechmaille et al. 2011a, Lorch et al. 2012, Raudabaugh and Miller 2013, Reynolds et al. 2015, Vanderwolf et al. 2016a, Lindner et al. 2017), as well as by its clonal mode of reproduction. If sex was the dominant mode of reproduction, a higher turnover of MLGs would be expected throughout the years due to the generation of new MLGs following recombination, which is not what we observe in Eldena and Balabanova dupka. The apparent higher turnover observed in Ivanova voda is most likely explained by the lower number of single spore isolates obtained from this cave and spring flooding potentially washing away or killing fungal spores (Suppl. materials 2, 4). Nevertheless, considering the genetic machinery needed for sexual recombination is present in *P.destructans* (Palmer et al. 2014) and that the two mating types co-occur within the same site, sexual reproduction is still possible and more studies are needed on the subject.

On the other hand, the population structure of *P.destructans*, based on different rooms of the same hibernaculum, had never been studied before and the absence of significant genetic differentiation is consistent with the ecology of hibernating bat species. In autumn, when bats arrive at their hibernaculum, they often engage in mating or other social interaction, flying around, landing and crawling on different places/rooms of the roost walls, the so-called swarming behaviour. Elevated activity of bats within the site is also observed in spring, when the animals prepare for moving out and can hang in different places across the hibernaculum. Even in winter, bats regularly interrupt their torpor bouts (Blažek et al. 2019) and may engage in grooming or contact other individuals or areas of the roost environment (Bartonička et al. 2017, Hoyt et al. 2021). Together, such types of behaviour are expected to lead to ample movement of the fungus across hibernacula used by bats (Bücs et al. 2023). Yet, we cannot exclude the role of other factors, such as air currents (Kokurewicz et al. 2016) or movement of arthropods (Bastian et al. 2009, Lučan et al. 2016, Vanderwolf et al. 2016b, Zahradníková et al. 2017).

Altogether, our results show that *P.destructans* populations are not genetically differentiated within a site, suggesting ample pathogen movement. This does not rule out the possibility that certain factors (different microclimates or the segregation of bat species within a site) could act as barriers for *P.destructans* or lead to the selection of certain pathogen genotypes. However, even if such barriers/selection pressures exist, it seems that bat movement within a site is sufficient to mask their effects.

### Significant P.destructans genetic differentiation at large scale

In agreement with our expectation, we detected significant allelic differentiation in *P.destructans* populations and we did not find a single pathogen MLG shared between sites for the full duration of our study. Although our number of sites is low (n = 3), it is worth noting that our results are in complete agreement with the results of Fischer et al. (2022) who included a higher number of sites (n = 9), closely situated to each other and without apparent barriers to movement for the bat hosts. These results could appear to contradict research from North America where population structure is limited and MLGs can be shared over large distances (Forsythe et al. 2021). However, the limited population structure observed in North America is most likely the result of the recent expansion (colonisation) rather than a signal of ongoing extensive gene flow between established *P.destructans* populations (see also Fischer et al. 2022). Besides, due to the founder effect, whereby genetic richness in the invasive population of a species is reduced due to the introduction of a single or just a few individuals, genetic diversity of *P.destructans* in North America is magnitudes lower in comparison to Eurasia (Drees et al. 2017a), which reduces the power of genetic markers to detect population differentiation at such short timescales. To overcome this issue, Thapa et al. (2021) used the fast-evolving mycovirus PdPV-pa as a proxy to infer the *P.destructans* population structure and found very strong differentiation across the studied region in North America. Although there are different factors that can decouple the mycovirus (PdPV-pa) population structure from its host (*P.destructans*) population structure (Mazé-Guilmo et al. 2016), the specificities of the current system point towards a situation where both structures should be correlated. Indeed, PdPV-pa seems to specifically infect *P.destructans* (Ren et al. 2020), all North-American *P.destructans* isolates seem to be infected with PdPV-pa (Thapa et al. 2016, Ren et al. 2020, Thapa et al. 2021) and mycoviruses lack an extracellular life stage and are transmitted only through cell division, sporulation or cell fusion (Pearson et al. 2009, Son et al. 2015, Kotta-Loizou 2021). Taken together, these studies (Thapa et al. 2021, Fischer et al. 2022; the present study) strongly suggest that once *P.destructans* populations have established, the successful establishment of newly-arrived pathogen MLGs is rare in comparison to *in situ* recruitment (see also Fischer et al. 2022). Thus, a detailed characterisation of the continental population structure of *P.destructans* across Europe may predict its population structure in North America after reaching equilibrium.

### P.destructans genetic diversity between and within sites

Our results confirmed the expectation that *P.destructans* genotypic richness at different sites in Europe varies within the same order of magnitude, pointing to the presence of up to 400 different MLGs in a single hibernaculum, possibly even more. The differences in *P.destructans* genetic diversity between Balabanova dupka and Ivanova voda on the one hand and Eldena on the other hand can be due to several factors such as: type of roost (natural roosts are larger and older and provide more diversity of microhabitats and microbial communities, as well as more organic matter); bat colony size (thousands in the caves vs. hundreds in Eldena); usage by bats (possibly hundreds to thousands of years in the caves vs. several decades in Eldena); geographic location (biological diversity is higher in the lower in comparison to the higher latitudes); or possible environmental growth of *P.destructans*. Yet, our data do not allow us to identify the most significant of those.

No hibernacula have been intensively surveyed in North America to allow the calculation of genotypic richness within an individual site, but patterns observed across many sites with a limited number of isolates characterised and a reduced number of loci (9 versus 18 in the present study) suggest a much reduced genotypic richness with MLGs shared across much larger distances than in Europe (e.g. Forsythe et al. 2021 vs. Fischer et al. 2022). Consistently, the number of alleles per locus is much reduced in North America compared to Europe (Table S2 in Forsythe et al. 2021 vs. Table S1 in Fischer et al. 2022 and Suppl. material 6 herein; Table 2 in Drees et al. 2017a). This is consistent with the population bottleneck, resulting from the pathogen’s recent introduction to North America and subsequent geographical expansion (Leopardi et al. 2015, Drees et al. 2017a, Drees et al. 2017b).

Within the same site, we found shared MLGs between walls and bats and consistently higher genotypic richness of *P.destructans* on walls compared to bats. Thus, our results corroborate the hypothesis tested in Fischer et al. (2022) stating that hibernaculum walls represent the environmental reservoir of the pathogen and the initial source of bat infection (see also Hicks et al. 2023).

### Genetically-diverse infections in bats

Infections with multiple MLGs were prevalent in bats, with more than 80% of the bat swabs harbouring multiple *P.destructans* MLGs. However, we only considered three fungal isolates per swab and the number of *P.destructans* spores that infect a bat at the start of hibernation is estimated to be roughly between 50 and 500 (Fischer et al. 2022). Thus, it is expected that practically every bat infected with *P.destructans*, at least within its native range, will be infected with numerous pathogen MLGs. To our knowledge, our study is the first to investigate multiple infections in *P.destructans*, a finding that is important to consider for two reasons. First, significant variation in pathogenicity can exist even between closely-related variants of the same fungal pathogen species (Raabe 1972). Second, due to various interactions between pathogen MLGs, genetically diverse infections (co-infections) often behave differently from clonal ones. For example, competition for the host resources between different MLGs can lead to infection suppression and protection against superinfection (Mercereau-Puijalon 1996, Tanner et al. 1999) or, on the contrary, increase pathogen burden by stimulating pathogens to occupy broader niche space or raising the cost of the immune response (Taylor et al. 1998). Additionally, interactions between pathogen MLGs can alter the density of more or less virulent MLGs (Read and Taylor 2001) and possibly represent a powerful determinant for pathogen evolution and disease epidemiology (Susi et al. 2015). In some cases, hosts co-infected with multiple pathogen MLGs exhibit more severe disease symptoms and more intense transmission of the pathogen as shown in the host plant *Plantagolanceolata* and its fungal parasite *Podosphaeraplantaginis* (Susi et al. 2015). In other cases, genetically diverse infections seem to reduce pathogen load as in the snail *Gasterosteusaculeatus* and its trematode parasite *Diplostomumpseudospathaceum* (Rauch et al. 2007). Furthermore, the characteristics of multiple infections might be different depending on the degree of relatedness between pathogen MLGs with competitive exclusion of distant MLGs and tolerance towards closely-related MLGs (López-Villavicencio et al. 2007). As a consequence, it would be interesting to study how infections with multiple *P.destructans* MLGs influence the outcome of the disease. Knowledge on the pathogen’s genetic richness within individual hibernacula and on individual bats (presented here), combined with information on *P.destructans* inoculum size on bats at the start of hibernation (presented in Fischer et al. (2022)), are of paramount importance to address these questions as they provide an estimate of the number of different MLG interacting on individual bats.

## Conclusion

We discovered no significant genetic differentiation in *P.destructans* population within sites contrasting with the strong genetic differentiation observed between sites. This indicates that the rate of pathogen movement is magnitudes higher within compared to between hibernacula and underlines the importance of studies investigating spatial and temporal changes at individual sites to better understand the intricacies of host-pathogen interactions. Additionally, we highlight the considerable genotypic richness of the pathogen within any of our study sites, which, in turn, leads to a high frequency of multiple infections in bats with potentially important biological consequences. Altogether, our results not only advance fundamental knowledge on *P.destructans* and WND, but also provide critical information to design studies and suggest novel directions for future research.

## Supplementary Material

D39C5C15-73CC-533E-9E24-C273FB9214FE 10.3897/BDJ.11.e109848.suppl1 Supplementary material 1 Study sites Data type picture Brief description Geographic location of our study sites. Balabanova dupka and Ivanova voda are natural karst caves only accessible with caving equipment and Eldena is an artificial hibernaculum, a disused cellar. File: oo_877262.png https://binary.pensoft.net/file/877262 Nicola Fischer

06924900-A940-5692-BD11-8A1DB75CFD06 10.3897/BDJ.11.e109848.suppl2 Supplementary material 2 Sampling locations Bulgaria Data type image (pdf) Brief description Sampling locations (rooms) in relevance to the hibernating bat colonies in the two studied Bulgarian caves. In Balabanova dupka, the entire colony of *Myotismyotis*/*blythii* hibernates in Room 1. Bats are found occasionally in spring and autumn in Rooms 2 & 3. The map is modified from Georgiev et al. (2016). In Ivanova voda, the colony of *Myotismyotis*/*blythi* hibernates mostly in Rooms 2 & 3 and the colony of *Myotiscapaccinii* hibernates mostly in Room 4 and 5, above a large lake. The cave can flood in spring, the water reaching up to Room 3. The original map was made by S. Adreeev and H. Delchev (1962). File: oo_877233.pdf https://binary.pensoft.net/file/877233 Violeta Zhelyazkova

E8410062-559A-584A-864A-2EA15622175F 10.3897/BDJ.11.e109848.suppl3 Supplementary material 3 Sampling locations Germany Data type image Brief description Sampling locations (rooms) in the artificial hibernaculum Eldena in Germany. Here, *Myotisdaubentonii*, *M.nattereri* and *M.myotis* hibernate. R stands for Room. Bats hibernate in all rooms although in different numbers. Samples were collected in all rooms, except rooms 1 and 12. File: oo_877234.png https://binary.pensoft.net/file/877234 Sebastien J. Puechmaille

78E2CD25-4FF7-5101-AEEE-DC4D19872F37 10.3897/BDJ.11.e109848.suppl4 Supplementary material 4 Sample summary per season Data type table Brief description Summary of the numbers of swab samples, single spore isolates (SSIs) and multilocus genotypes (MLGs) of *P.destructans* obtained from bats and from hibernacula walls in the study sites divided by season. File: oo_877235.docx https://binary.pensoft.net/file/877235 Violeta Zhelyazkova

CC5EE1B2-26AF-51B7-A1F8-E29268B06E97 10.3897/BDJ.11.e109848.suppl5 Supplementary material 5 Sample summary per room Data type table Brief description Summary of the number of swab samples, single spore isolates (SSIs) and multilocus genotypes (MLGs) of *P.destructans* obtained from bats and from hibernacula walls in the study sites divided by rooms. File: oo_877240.docx https://binary.pensoft.net/file/877240 Violeta Zhelyazkova

EAFB79A7-095D-5F9D-8576-DF54F2E7A100 10.3897/BDJ.11.e109848.suppl6 Supplementary material 6 Allelic richness Data type table Brief description Allelic richness per locus in the three study sites. Calculations were performed on clone corrected data after single spore isolates with missing data were removed. File: oo_877247.docx https://binary.pensoft.net/file/877247 Violeta Zhelyazkova

52C4B6E7-3D40-5228-B07E-9A9F17B3B27D 10.3897/BDJ.11.e109848.suppl7 Supplementary material 7 Heterogeneity test Data type graph Brief description Outputs of the heterogeneity test developed by Puechmaille & Petit (2007) for the three study sites. This analysis is used to test the assumption of the capture-mark-recapture (CMR) model that each MLG has the same probability of being sampled. Filled circles show the model distribution of captures or singles spore isolates (SSIs) obtained per individual multilocus genotype (MLG) under the assumption that each MLG has the same probability of being sampled (the population is homogenous). Empty circles show the highest probability density of the population size estimate (HPD95%). Triangles show the observed distribution of obtained SSIs per individual MLG. File: oo_877258.pdf https://binary.pensoft.net/file/877258 Violeta Zhelyazkova, Sebastien Puechmaille

B539DE97-6736-5870-A9B3-EEEABCA70C2C 10.3897/BDJ.11.e109848.suppl8 Supplementary material 8 P.destructans genetic diversity per site
Data type table Brief description Comparison of *P.destructans* genetic diversity in the study sites when one single spore isolate only is considered per swab. Swab is the total number of swab samples collected from each site, both from bats and hibernacula walls; SSI is the total number of single spore isolates of *P.destructans* (= sample size); Allele is the mean number of alleles per locus; MLG is the total number of multilocus genotypes observed; eMLG is the number of expected multilocus genotype at the smallest shared sample size between the three sites (n = 255); Pop size is the estimated population size based on the CMR model; HPD 95% is the highest probability density of the population size estimate; M1 & M2 are the percentage of mating type MAT1_1 & MAT1_2, respectively. File: oo_877260.docx https://binary.pensoft.net/file/877260 Violeta Zhelyazkova

86BE61C1-7DA8-5390-B03E-A4BF7B41FDE2 10.3897/BDJ.11.e109848.suppl9 Supplementary material 9 P.destructans genetic diversity on bats and walls
Data type table Brief description Comparison of *P.destructans* genotypic diversity found on bats and walls in the study sites when one (top) or two (bottom) SSIs are considered per swab. Abbreviations are as defined for Supplement 5. For the analysis with two single spore isolates per swab, all swabs that had given only one single spore isolate were removed. As the smallest shared sample size is calculated for bats and walls within each individual site, the reported eMLG values should only be compared within sites. File: oo_877270.docx https://binary.pensoft.net/file/877270 Violeta Zhelyazkova

2061B029-7910-5B06-A05C-D7D59283F2F9 10.3897/BDJ.11.e109848.suppl10 Supplementary material 10 Dataset Data type data table Brief description This is the dataset combining microsatellite data on *P.destructans* from Bulgaria and Germany analysed in the present research. File: oo_877267.csv https://binary.pensoft.net/file/877267 Violeta Zhelyazkova, Nicola Fischer, Sebastien Puechmaille

345937A8-B698-5997-86CF-9B15E1D5AF97 10.3897/BDJ.11.e109848.suppl11 Supplementary material 11 R script Data type R script Brief description This is the R script used to analyse the data. File: oo_877269.R https://binary.pensoft.net/file/877269 Violeta Zhelyazkova, Nicola Fischer, Sebastien Puechmaille

## Figures and Tables

**Table 1.** Comparison of *P.destructans* genetic diversity in the three study sites. Swab is the total number of swab samples collected from each site, both from bats and hibernaculum walls; SSI is the total number of single spore isolates of *P.destructans* (= sample size); Allele is the mean number of alleles per locus; MLG is the total number of multilocus genotypes observed; eMLG is the number of expected multilocus genotype at the smallest shared sample size between the three sites (n = 255); Pop size is the estimated population size, based on the CMR model; HPD95% is the highest probability density of the population size estimate; M1 & M2 are the percentages of mating type MAT1_1 & MAT1_2, respectively.

	Swab	SSIs	Allele	MLG	eMLG	Pop size	HPD 95%	M1	M2
Balabanova dupka	172	608	12.5	301	170.5	377	352-401	68.5%	31.5%
Ivanova voda	74	255	14.6	165	165	274	233-317	70.2%	29.8%
Eldena	364	1062	6	149	77.3	150	150-151	42.4%	57.6%

**Table 2.** Comparison of *P.destructans* genotypic richness found on bats and walls in the study sites. Abbreviations are as defined for Table 1. (G-1)/(N-1) is the mean genotypic richness per swab, where G is the number of MLGs per swab and N is the number of single spore isolates (SSIs) per swab. As the smallest shared sample size is calculated for bats and walls within each individual site, the reported eMLG values should only be compared within sites.

		Swab	SSI	MLG	eMLG	(G-1)/(N-1)
Balabanova dupka	Bats	63	305	159	158.3	0.58
	Walls	109	303	196	196	0.94
Ivanova voda	Bats	40	180	101	55.1	0.54
	Walls	34	75	69	69	0.97
Eldena	Bats	286	788	118	76.5	0.61
	Walls	78	274	84	84	0.85
