# Supplementary figures and images for: Bat white-nose disease fungus diversity in time and space
Source: Biodivers Data J. 2024 Feb 2;12:e109848. doi: 10.3897/BDJ.12.e109848 (PMC10859861; doi:10.3897/BDJ.12.e109848)

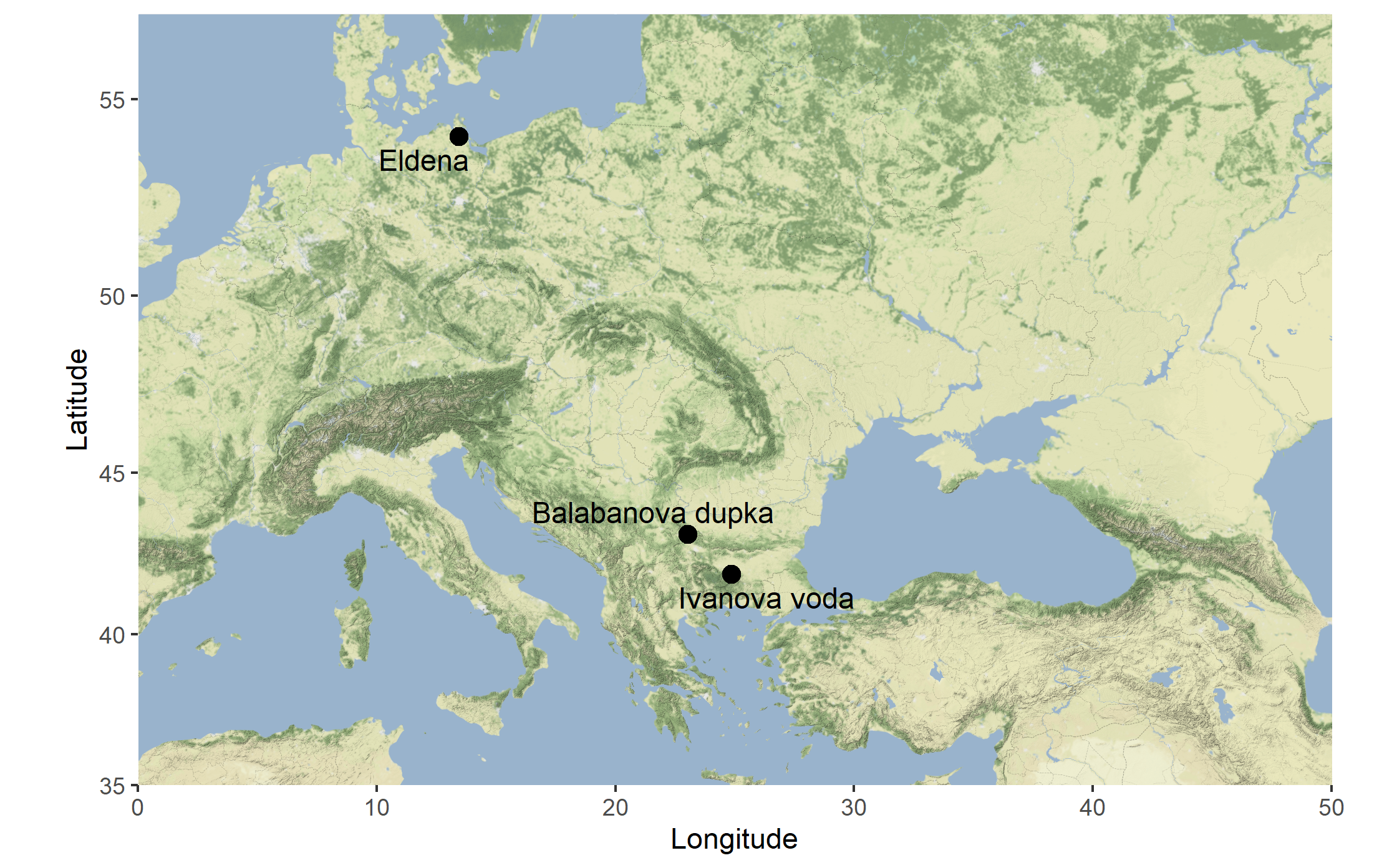

Supplement: Supplementary material 1 — Study sites [file bdj-12-e109848-s001.png]

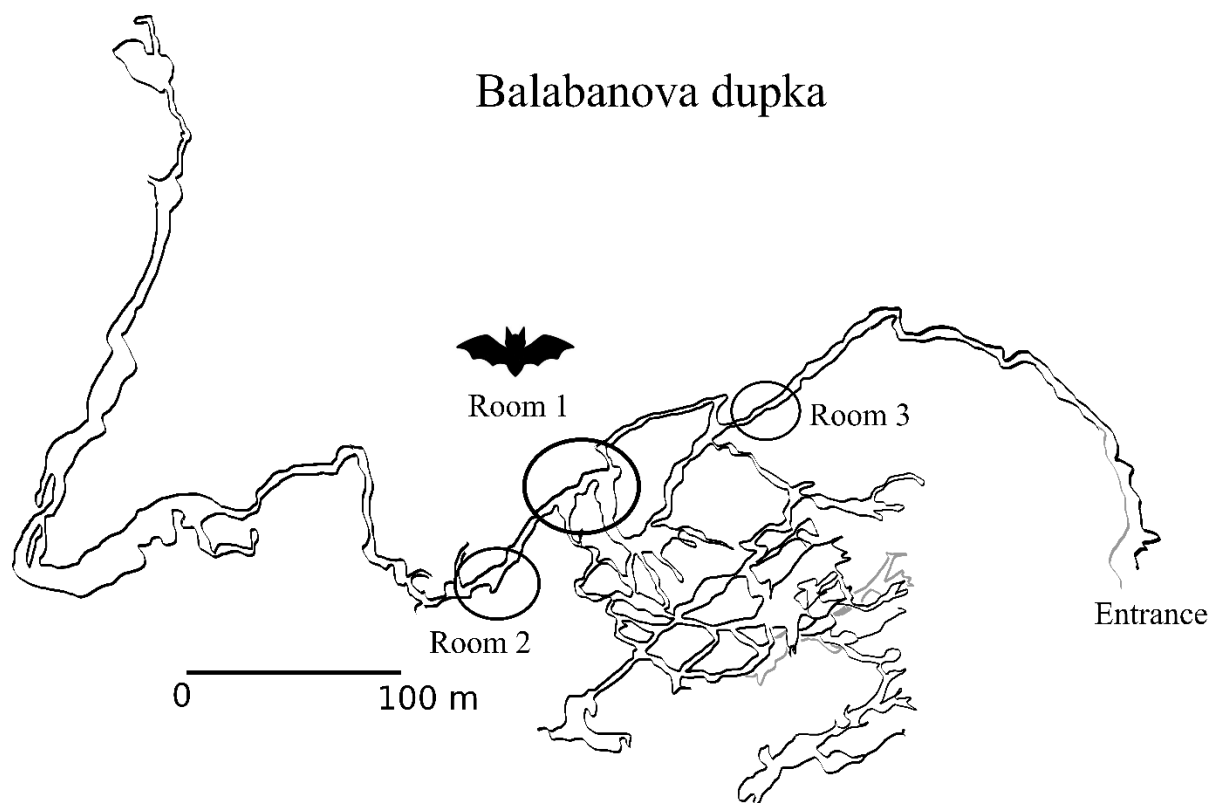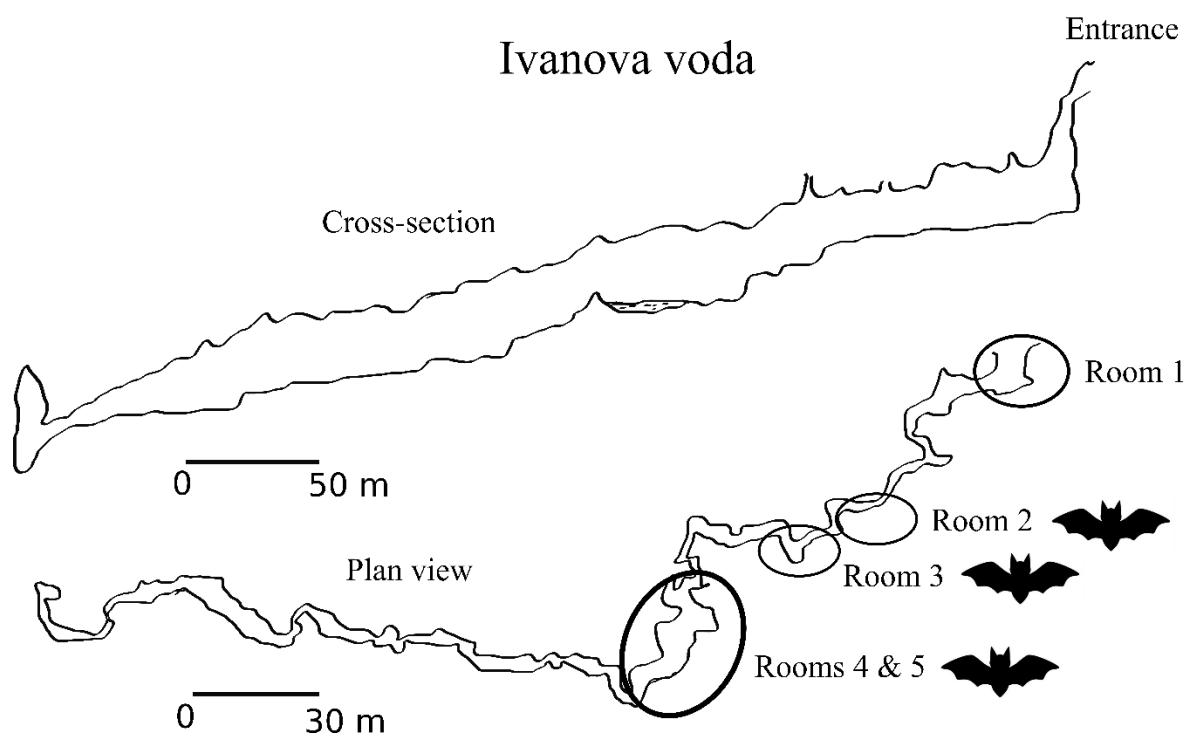

Supplement: Supplementary material 2 — Sampling locations Bulgaria [file bdj-12-e109848-s002.pdf]

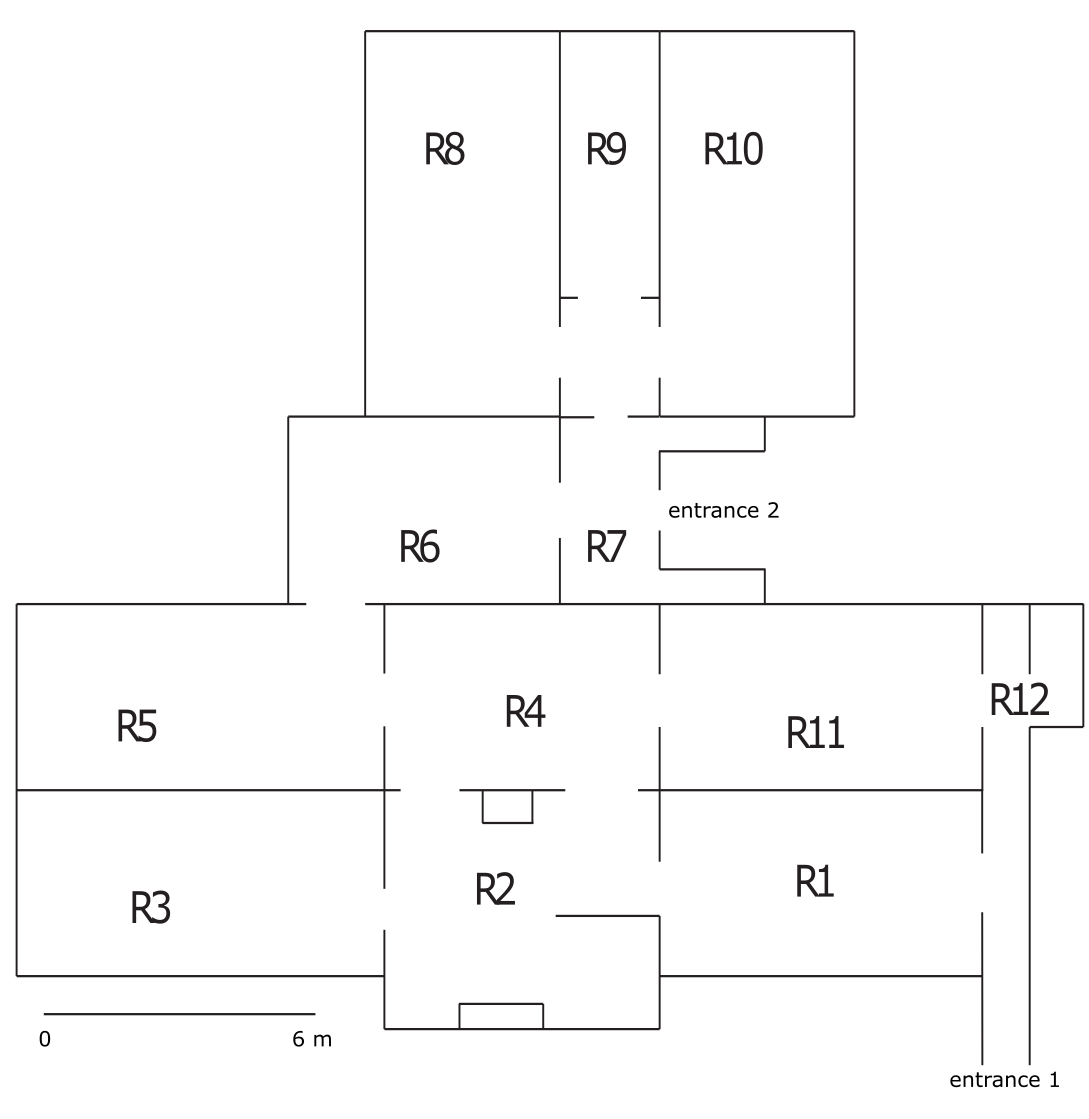

Supplement: Supplementary material 3 — Sampling locations Germany [file bdj-12-e109848-s003.png]

# Balabanova dupka

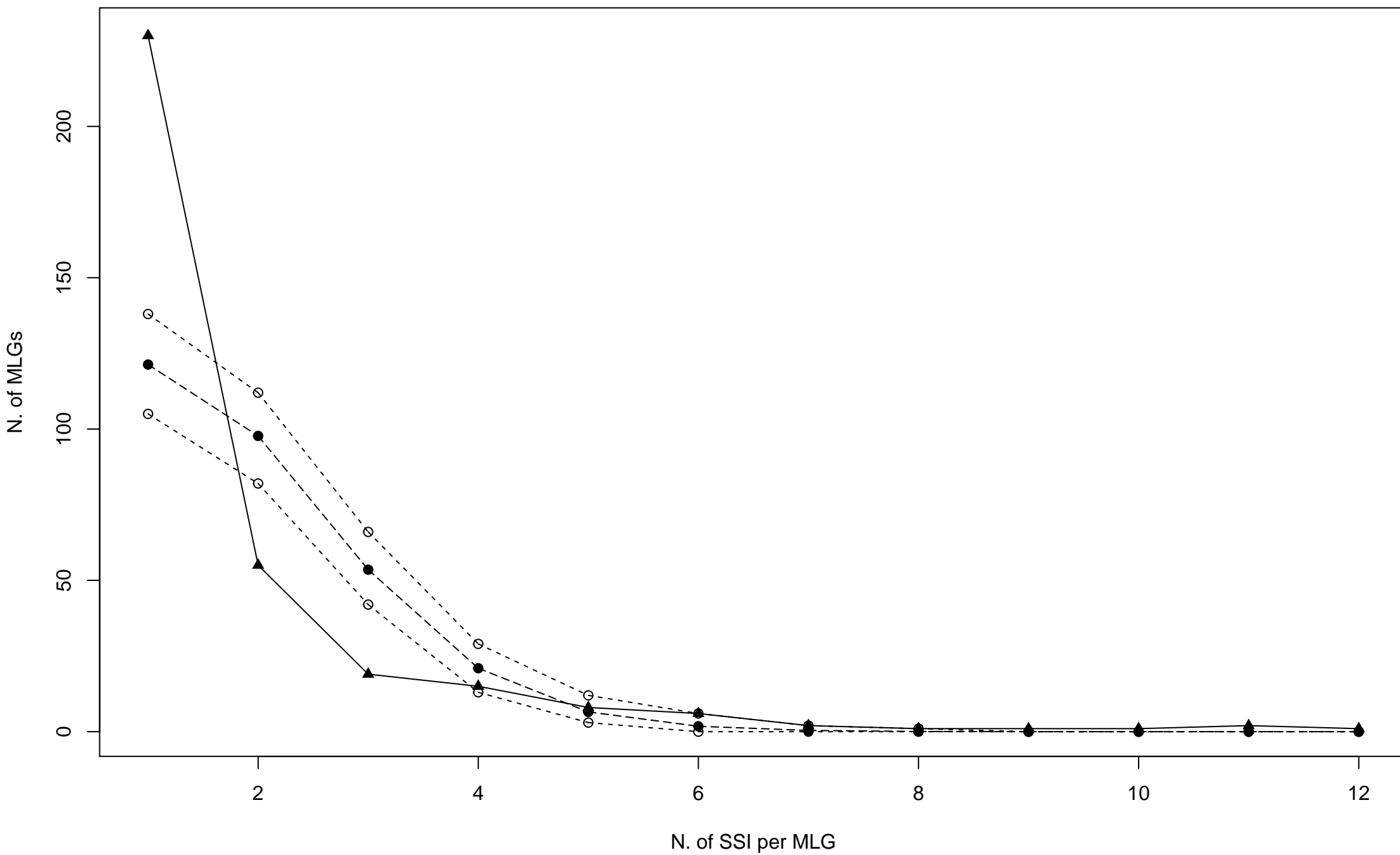

# Eldena

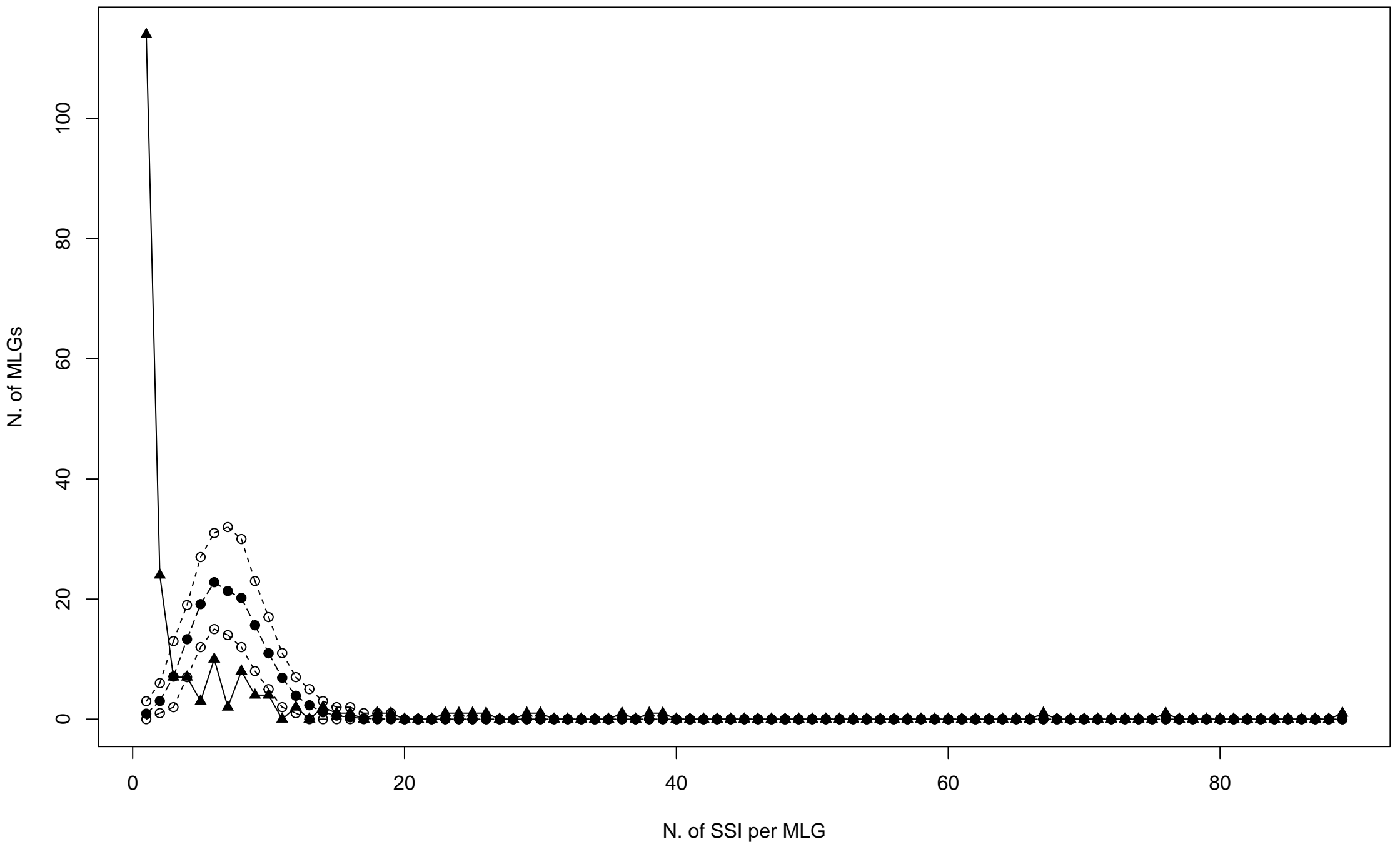

# Ivanova voda

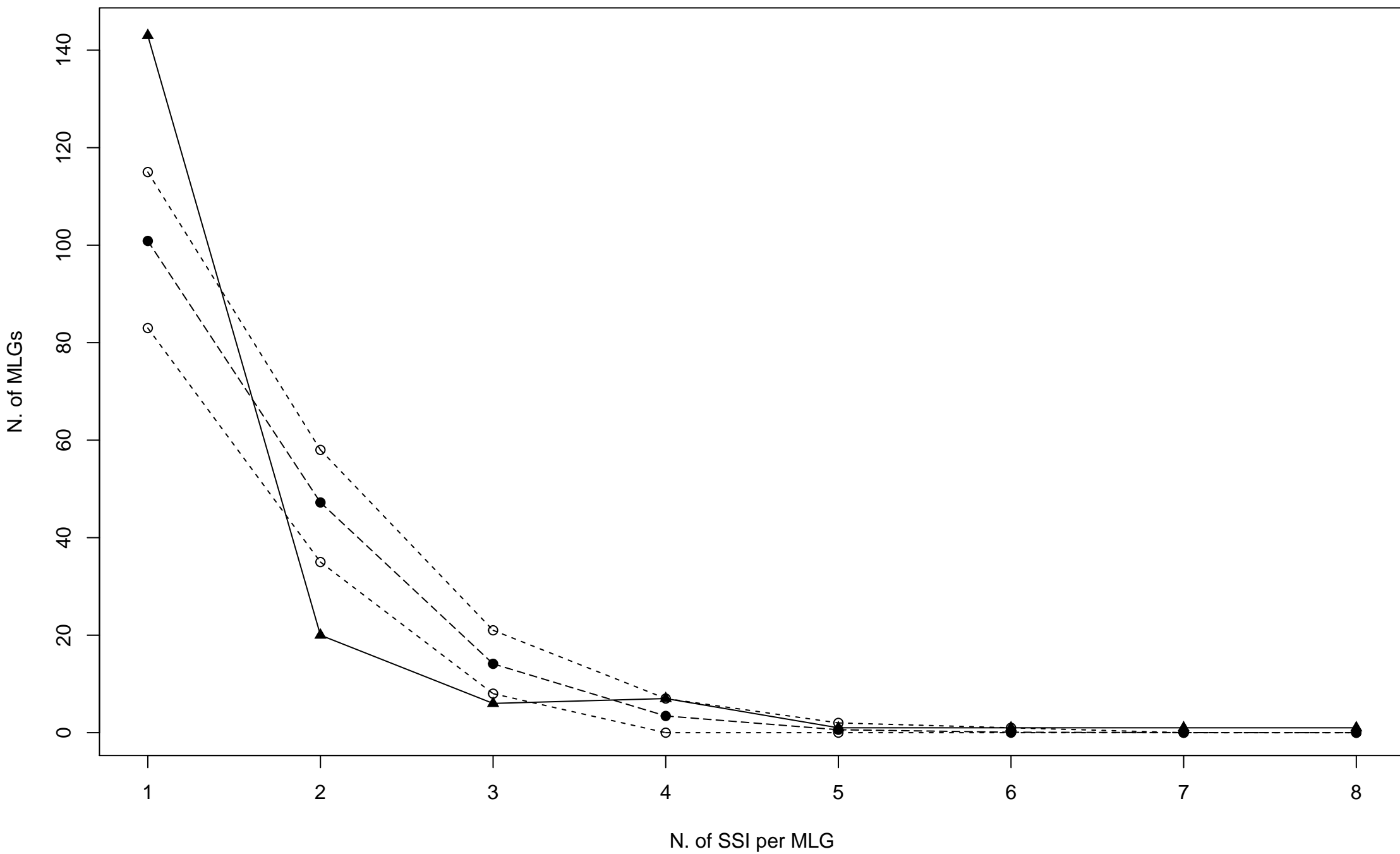

Supplement: Supplementary material 7 — Heterogeneity test [file bdj-12-e109848-s007.pdf]
