# Supplementary material for: Bat white-nose disease fungus diversity in time and space
Source: Biodivers Data J. 2024 Feb 2;12:e109848. doi: 10.3897/BDJ.12.e109848 (PMC10859861; doi:10.3897/BDJ.12.e109848)
Supplement: Supplementary material 4 — Sample summary per season [file bdj-12-e109848-s004.docx]

| Sampling season | Total swab | Bat swab | Wall swab | Total SSI | Bat SSI | Wall SSI | Total MLG | Bat MLG | Wall MLG |
| --- | --- | --- | --- | --- | --- | --- | --- | --- | --- |
| Balabanova dupka | | | | | | | | | |
| Spring 2015 | 23 | 21 | 2 | 97 | 90 | 7 | 62 | 58 | 6 |
| Spring 2017 | 34 | 11 | 23 | 99 | 48 | 51 | 72 | 35 | 44 |
| Autumn 2017 | 13 | 0 | 13 | 21 | 0 | 21 | 18 | 0 | 18 |
| Spring 2018 | 27 | 12 | 15 | 95 | 60 | 35 | 62 | 32 | 34 |
| Autumn 2018 | 19 | 0 | 19 | 37 | 0 | 37 | 36 | 0 | 36 |
| Spring 2019 | 56 | 19 | 37 | 259 | 107 | 152 | 151 | 58 | 113 |
| Ivanova voda | | | | | | | | | |
| Spring 2017 | 29 | 15 | 14 | 98 | 62 | 36 | 78 | 45 | 34 |
| Autumn 2017 | 1 | 0 | 1 | 3 | 0 | 3 | 3 | 0 | 3 |
| Spring 2018 | 8 | 5 | 3 | 33 | 25 | 8 | 20 | 12 | 8 |
| Autumn 2018 | 8 | 0 | 8 | 8 | 0 | 8 | 8 | 0 | 8 |
| Spring 2019 | 28 | 20 | 8 | 113 | 93 | 20 | 68 | 51 | 18 |
| Eldena | | | | | | | | | |
| Spring 2015 | 147 | 139 | 8 | 425 | 391 | 34 | 77 | 74 | 21 |
| Autumn 2015 | 16 | 0 | 16 | 39 | 0 | 39 | 29 | 0 | 29 |
| Spring 2016 | 40 | 31 | 9 | 118 | 80 | 38 | 44 | 34 | 22 |
| Autumn 2016 | 6 | 0 | 6 | 22 | 0 | 22 | 17 | 0 | 17 |
| Spring 2017 | 10 | 10 | 0 | 26 | 26 | 0 | 16 | 16 | 0 |
| Autumn 2017 | 16 | 0 | 16 | 55 | 0 | 55 | 30 | 0 | 30 |
| Spring 2018 | 61 | 53 | 8 | 183 | 149 | 34 | 45 | 37 | 19 |
| Autumn 2018 | 15 | 0 | 15 | 52 | 0 | 52 | 32 | 0 | 32 |
| Spring 2019 | 53 | 53 | 0 | 142 | 142 | 0 | 47 | 47 | 0 |
