# Supplementary material for: Bat white-nose disease fungus diversity in time and space
Source: Biodivers Data J. 2024 Feb 2;12:e109848. doi: 10.3897/BDJ.12.e109848 (PMC10859861; doi:10.3897/BDJ.12.e109848)
Supplement: Supplementary material 5 — Sample summary per room [file bdj-12-e109848-s005.docx]

| Room | Total swab | Bat swab | Wall swab | Total SSI | Bat SSI | Wall SSI | Total MLG | Bat MLG | Wall MLG |
| --- | --- | --- | --- | --- | --- | --- | --- | --- | --- |
| Balabanova dupka | | | | | | | | | |
| 1 | 110 | 56 | 54 | 411 | 273 | 138 | 217 | 144 | 105 |
| 2 | 55 | 0 | 55 | 165 | 0 | 165 | 129 | 0 | 129 |
| 3 | 7 | 7 | 0 | 32 | 32 | 0 | 23 | 23 | 0 |
| Ivanova voda | | | | | | | | | |
| 1 | 3 | 0 | 3 | 3 | 0 | 3 | 3 | 0 | 3 |
| 2 | 60 | 38 | 22 | 230 | 172 | 58 | 147 | 97 | 53 |
| 3 | 6 | 1 | 5 | 13 | 5 | 8 | 10 | 2 | 8 |
| 4 | 3 | 0 | 3 | 5 | 0 | 5 | 5 | 0 | 5 |
| 5 | 1 | 0 | 1 | 1 | 0 | 1 | 1 | 0 | 1 |
| NA | 1 | 1 | 0 | 3 | 3 | 0 | 3 | 3 | 0 |
| Eldena | | | | | | | | | |
| 2 | 12 | 12 | 0 | 35 | 35 | 0 | 16 | 16 | 0 |
| 3 | 7 | 7 | 0 | 19 | 19 | 0 | 9 | 9 | 0 |
| 4 | 4 | 4 | 0 | 12 | 12 | 0 | 3 | 3 | 0 |
| 5 | 11 | 11 | 0 | 28 | 28 | 0 | 15 | 15 | 0 |
| 6 | 12 | 11 | 1 | 28 | 27 | 1 | 15 | 15 | 1 |
| 7 | 1 | 0 | 1 | 4 | 0 | 4 | 3 | 0 | 3 |
| 8 | 89 | 60 | 29 | 257 | 162 | 95 | 71 | 48 | 47 |
| 9 | 83 | 66 | 17 | 249 | 183 | 66 | 70 | 59 | 37 |
| 10 | 107 | 79 | 28 | 318 | 215 | 103 | 76 | 59 | 43 |
| 11 | 11 | 9 | 2 | 31 | 26 | 5 | 16 | 14 | 3 |
| NA | 27 | 27 | 0 | 81 | 81 | 0 | 25 | 25 | 0 |
