# Supplementary material for: Bat white-nose disease fungus diversity in time and space
Source: Biodivers Data J. 2024 Feb 2;12:e109848. doi: 10.3897/BDJ.12.e109848 (PMC10859861; doi:10.3897/BDJ.12.e109848)
Supplement: Supplementary material 6 — Allelic richness [file bdj-12-e109848-s006.docx]

|  | Balabanova dupka | Ivanova voda | Eldena |
| --- | --- | --- | --- |
| Locus name | Number of alleles | | |
| Pd1 | 25 | 28 | 14 |
| Pd2 | 64 | 49 | 8 |
| Pd3 | 13 | 11 | 3 |
| Pd4 | 20 | 18 | 13 |
| Pd5 | 5 | 31 | 13 |
| Pd6 | 6 | 5 | 3 |
| Pd7 | 14 | 12 | 5 |
| Pd9 | 5 | 8 | 4 |
| Pd10 | 3 | 7 | 3 |
| Pd11 | 8 | 14 | 4 |
| Pd12 | 6 | 5 | 2 |
| Pd13 | 10 | 10 | 13 |
| Pd14 | 10 | 14 | 5 |
| Pd17 | 7 | 8 | 3 |
| Pd19 | 11 | 12 | 5 |
| Pd21 | 7 | 7 | 3 |
| Pd22 | 9 | 14 | 4 |
| Pd23 | 2 | 9 | 3 |
| Mean | 12.5 | 14.6 | 6 |
