# Supplementary material for: Bat white-nose disease fungus diversity in time and space
Source: Biodivers Data J. 2024 Feb 2;12:e109848. doi: 10.3897/BDJ.12.e109848 (PMC10859861; doi:10.3897/BDJ.12.e109848)
Supplement: Supplementary material 8 — P.destructans genetic diversity per site [file bdj-12-e109848-s008.docx]

|  | Swab | SSI | Allele | MLG | eMLG | Pop size | HPD  95% | M1 | M2 |
| --- | --- | --- | --- | --- | --- | --- | --- | --- | --- |
| Balabanova dupka | 172 | 172 | 11.2 | 137.4 | 66.4 | 395.6 | 282-516 | 68.7% | 31.3% |
| Ivanova voda | 74 | 74 | 11.6 | 67.2 | 67.2 | 569.2 | 203-1082 | 73.8% | 26.2% |
| Eldena | 364 | 364 | 5.8 | 93.1 | 39.3 | 96.2 | 94-99 | 41.9% | 58.1% |
