# Supplementary material for: Bat white-nose disease fungus diversity in time and space
Source: Biodivers Data J. 2024 Feb 2;12:e109848. doi: 10.3897/BDJ.12.e109848 (PMC10859861; doi:10.3897/BDJ.12.e109848)
Supplement: Supplementary material 9 — P.destructans genetic diversity on bats and walls [file bdj-12-e109848-s009.docx]

|  |  | Swab | SSI | MLG | eMLG |
| --- | --- | --- | --- | --- | --- |
| One SSI per swab | | | | | |
| Balabanova dupka | Bats | 63 | 63 | 57.3 | 57.3 |
|  | Walls | 109 | 109 | 92.8 | 57.1 |
| Ivanova voda | Bats | 40 | 40 | 36.6 | 31.6 |
|  | Walls | 34 | 34 | 33.2 | 33.2 |
| Eldena | Bats | 286 | 286 | 80.6 | 40.1 |
|  | Walls | 78 | 78 | 42.1 | 42.1 |
| Two SSIs per swab | | | | | |
| Balabanova dupka | Bats | 63 | 126 | 93.5 | 93.5 |
|  | Walls | 64 | 128 | 100.1 | 98.8 |
| Ivanova voda | Bats | 39 | 78 | 58.6 | 28.2 |
|  | Walls | 16 | 32 | 31.3 | 31.3 |
| Eldena | Bats | 268 | 536 | 104.4 | 51.9 |
|  | Walls | 61 | 122 | 52.7 | 52.7 |
